# Modulating membrane fusion through the design of fusogenic DNA circuits and bilayer composition†

**DOI:** 10.1039/d2sm00863g

**Published:** 2022-08-17

**Authors:** Miguel Paez-Perez, I. Alasdair Russell, Pietro Cicuta, Lorenzo Di Michele

**Affiliations:** Molecular Sciences Research Hub, Department of Chemistry, Imperial College London Wood Lane London W12 0BZ UK l.di-michele@imperial.ac.uk; fabriCELL, Imperial College London Wood Lane London W12 0BZ UK; Cancer Research UK Cambridge Institute, University of Cambridge Cambridge CB2 0RE UK; Biological and Soft Systems, Cavendish Laboratory, University of Cambridge Cambridge CB3 0HE UK ld389@cam.ac.uk

## Abstract

Membrane fusion is a ubiquitous phenomenon linked to many biological processes, and represents a crucial step in liposome-based drug delivery strategies. The ability to control, ever more precisely, membrane fusion pathways would thus be highly valuable for next generation nano-medical solutions and, more generally, the design of advanced biomimetic systems such as synthetic cells. In this article, we present fusogenic nanostructures constructed from synthetic DNA which, different from previous solutions, unlock routes for modulating the rate of fusion and making it conditional to the presence of soluble DNA molecules, thus demonstrating how membrane fusion can be controlled through simple DNA-based molecular circuits. We then systematically explore the relationship between lipid-membrane composition, its biophysical properties, and measured fusion efficiency, linking our observations to the stability of transition states in the fusion pathway. Finally, we observe that specific lipid compositions lead to the emergence of complex bilayer architectures in the fusion products, such as nested morphologies, which are accompanied by alterations in biophysical behaviour. Our findings provide multiple, orthogonal strategies to program lipid-membrane fusion, which leverage the design of either the fusogenic DNA constructs or the physico/chemical properties of the membranes, and could thus be valuable in applications where some design parameters are constrained by other factors such as material cost and biocompatibility, as it is often the case in biotechnological applications.

## Abstract 2

Control over lipid-membrane fusion is valuable in nanomedicine and synthetic biology. Here we provide guiding principles to program it by using fusogenic DNA nanostructures and exploring the effect of lipid composition on fusion efficiency.

## Graphical abstract

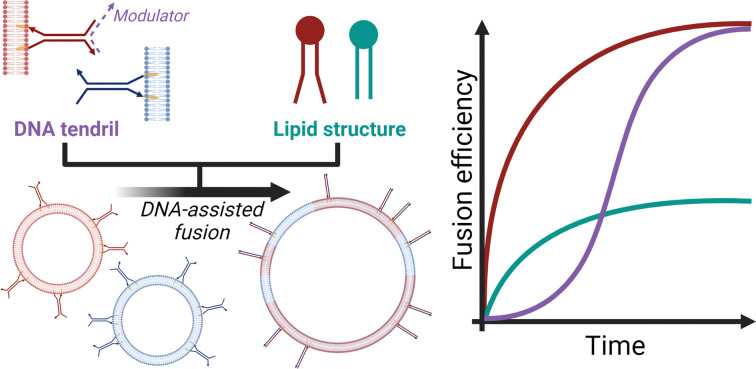

## 1 Introduction

Membrane fusion is a crucial process underpinning many biological phenomena, such as cargo transport between cellular compartments (*e.g.* endocytosis and exocytosis), cell division, communication, viral infection and lipid homeostasis.^1–3^ In addition, fusion of lipid bilayers has attracted a significant interest in biomedical research, as it plays a key role in liposomal-based drug delivery,^4–8^ cell transfection,^9,10^ the creation of artificial bioreactors^11–13^ or the development of synthetic cells capable of controlled product release,^14,15^ division and growth.^16^ Owing to this biological and biotechnological relevance, new strategies to rationally design lipid-membrane fusion pathways would be highly valuable.

Fusion between lipid bilayers is a two-step process.^1^ In the first step, the membranes must be brought into close proximity, overcoming repulsive electrostatic and steric forces, so that the leaflets from opposing bilayers can interact. Subsequently, in a second step, the lipid leaflets are destabilised and lipid molecules are transferred between the two bilayers. This exchange triggers the formation of a highly curved intermediate – the hemifusion stalk and diaphragm – which then expands until the two bilayers become fully merged.^1,17^ In order to rationally design lipid-fusion pathways, both these processes need to be controlled.

In nature, the process of overcoming the electrostatic and steric repulsion between lipid bilayers is regulated by the SNARE family of proteins. These proteins consist of a hydrophobic C-terminal domain embedded into the lipid membrane and a hydrophilic N-terminal domain containing the recognition motif. Upon interaction between SNARE proteins connected to opposing membranes, the SNARE complex forms through zipping up of the recognition motifs, which in turn brings the bilayers within close proximity and induces membrane fusion.^18^ SNARE proteins have inspired the development of synthetic fusogenic machineries, which mimic the response of the natural proteins but unlock greater opportunities for programability. Among these, are solutions based on synthetic nucleic acid devices, constructed using the principles of DNA nanotechnology.^19^ In this approach DNA motifs are anchored to the surface of synthetic membranes (typically liposomes) through hydrophobic modifications, including cholesterol, tocopherol and lipids.^20–26^ Zipping between complementary strands anchored on the surface of two different membranes then triggers their fusion,^21,27–29^ the efficiency of which has been found to depend on both DNA-nanostructure design and composition and lateral organisation of the bilayers.^27,28,30^ For example, membrane fusion was maximised when DNA zippers were equipped with two hydrophobic anchors, rather than a single one, reportedly because of the higher stability of the DNA–vesicle interaction,^30^ and when minimising the length of the linker connecting the hydrophobic moiety (cholesterol) to the DNA.^31^ Additionally, the extent of DNA-induced lipid mixing was shown to be impacted by the incorporation of cholesterol and phosphatidylethanolamine (PE) lipids^28^ or by changes in the lateral organisation of the membrane.^27^

Membrane composition dictates bilayer properties such as thickness, rigidity or spontaneous curvature, which in turn determine the energy cost for the formation of the highly-curved hemifusion stalk intermediate – the stabilisation of which is our second design challenge. In order for this structure to be formed, lipid composition must be tightly controlled.^32,33^ For example, lipids with saturated, longer hydrocarbon chains, or with charged headgroups, increase the free-energy cost for stalk formation, while the presence of lipids with small headgroups and Type II character (such as 1,2-dioleoyl-*sn-glycero*-3-phosphoethanolamine, DOPE) favour the formation of the fusion intermediate. In addition, changing the lipid composition can modulate the lateral organisation of the membrane, leading to the emergence of lipid domains with distinct biophysical properties.^34^ At the phase boundaries, membrane fusion is facilitated by the structural and chemical mismatch between the two domains.^27,35–37^

In this article we propose strategies to engineer lipid-membrane fusion, which address both key stages in the fusion pathway. First, we introduce alternative fusogenic DNA nanodevices, dubbed tendrils, which improving on previous implementations enable the conditional activation of the fusogenic pathway and its modulation thanks to competitive interactions with soluble components. In the second part of the article, we explore the effect of membrane composition on fusion efficiency, highlighting correlations between the latter, the hypothesised stability of the fusion intermediate, and key biophysical observables such as membrane curvature, thickness and area-per-lipid. Finally, we note that fusion between vesicles of specified compositions leads to the emergence of uncommon morphologies, highlighting the value of DNA-mediated fusion as a means of controlling the structure of lipid constructs. Taken together, our findings expand the existing toolkit for engineering membrane-fusion with orthogonal design principles that leverage either dynamic DNA nanotechnology or the physico/chemical properties of the membranes, and could thus be valuable in applications where some design parameters are constrained by other factors such as material cost and biocompatibility, as it is often the case in biotechnological applications.

## 2 Results

### 2.1 Fusion assay design

**Fig. 1:**
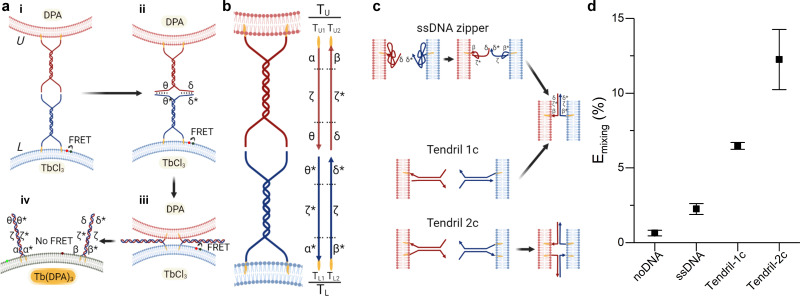
Improving the performance of DNA-mediated membrane fusion with tendril architectures. (a) Overall membrane-fusion process: (i) initially, FRET-labelled (L, blue, functionalised with *T*_L_ tendrils) and unlabelled (U, red, functionalised with *T*_U_ tendrils) vesicles encounter in solution. (ii) Overhangs in the DNA tendrils first link L and U liposomes. (iii) A four-way branch migration process reduces the distance between bilayers, bringing them within close distance and favouring the formation of the hemifusion stalk. (iv) Eventually, the fusion process may lead to the complete mixing of the membranes and interior contents of the L and U vesicles. Fusion progress is tracked through the decrease in FRET efficiency caused by the increase in distance between the FRET pair (initially present on L liposomes) upon fusion of L with U bilayers. Alternatively, L and U liposomes may be loaded with two compounds – TbCl_3_ and DPA – that when combining to form Tb(DPA) _3_ increase their fluorescence, indicating content mixing. (b) Schematic showing the single-stranded components constituting tendrils *T*_L_ and *T*_U_, each subdivided in three domains. The functionalisation of these structures with cholesterol moieties enables their stable binding to lipid membranes. (c) Expected interactions between lipid bilayers functionalised with ssDNA zippers or dsDNA tendrils decorated with one (1c) or two (2c) cholesterol anchors. (d) Normalised FRET efficiency *E*_mixing_, used as a proxy for membrane fusion and recorded 30 minutes after exposing U to L liposomes. A significant improvement in fusion efficiency is observed with the complete, double cholesterolised tendril constructs compared to the single-cholesterol version and the simpler ssDNA zipper design. See Tables S1 and S2 (ESI†) for ssDNA sequences and strands used for each DNA construct.

Fusion efficiency was evaluated in binary samples of large unilamellar vesicles (LUVs, nominal diameter ∼ 100 nm), using a lipid mixing assay based on Förster Resonant Energy Transfer (FRET),^30,38^ depicted in Fig. 1a and detailed in the ESI† methods. Briefly, one liposome population, L, was labelled with NBD-PE (donor) and rhodamine-PE (acceptor) headgroup-modified lipids, while the second population (U) was unlabelled. The mole fraction of labelled lipids was chosen so that FRET would efficiently occur on liposomes L. Upon fusion of L with U vesicles, the average NBD-rhodamine distance would increase, causing a decrease in FRET efficiency. The FRET efficiency, *E*_mixing_, normalised with respect to the “infinite dilution” value obtained by detergent solubilisation, was thus used as a proxy to track L–U fusion. We note that this observable is not representative of complete fusion, which requires content mixing confirmation, and does not provide an absolute quantification of the degree of lipid mixing (see ESI† methods for discussion). However it is a well-established method, which allows accurate comparisons between different fusogenic conditions.^30,38,39^ In selected experiments, the lipid-fusion assay was complemented with a content mixing assay whereby L and U liposomes were loaded with TbCl_3_ and DPA (dipicolinic acid), respectively, which upon mixing form the fluorescent Tb(DPA)_3_ complex^40^ (Fig. 1a).

In all experiments, the composition of U liposomes was kept unchanged as 50/25/25 DOPC/DOPE/Chol, selected as the gold standard fusogenic mixture based on previous reports.^21,28,31,41,42^ The presence of the Type II lipid DOPE and cholesterol is known to facilitate the membrane fusion through an increase of curvature stress, the promotion of the highly-curved inverted hexagonal phase (*H*_II_), and the reduction of the energy required to dehydrate the membrane and facilitate a close bilayer contact.^33,43,44^ The composition of L vesicles was varied depending on the specific experiment, as discussed below. L and U vesicles were were functionalised with the fusogenic DNA constructs after extrusion, as detailed in the ESI† methods.

### 2.2 DNA tendrils are active regulators of membrane fusion

In this section, we present an alternative design for DNA-based fusogenic nanostructures, dubbed tendrils, which enable modulation of fusion rates conditional to the presence and concentration of regulatory DNA elements in solution. Similar devices were recently adopted by (some of) the authors in the context of electrically induced fusion,^45^ but their performance for spontaneous fusion has not been studied before. For experiments presented in this section, the composition of L vesicles was kept identical to U liposomes.

Traditional implementations of fusogenic DNA nanostructures consist of a single DNA strand (ssDNA) anchored to the lipid bilayer through a hydrophobic moiety, such as cholesterol.^21,28,29^ However, ssDNA has been shown to collapse onto the bilayer surface owing to favourable interactions with PE headgroups,^46^ while the single cholesterol anchor prevents a stable insertion in the bilayer.^30,47^ Both these features could have detrimental effects on fusogenic activity of these DNA nanostructures.^30^

The DNA tendril design, shown in Fig. 1b, counters both these effects. Two constructs were prepared, *T*_L_ and *T*_U_ to be grafted on opposing L and U vesicle populations, respectively. The devices feature a rigid 21 base-pair (bp), double-stranded DNA (dsDNA) stem (spacer),^48^ which does not collapse onto the membrane, making the tendrils more accessible.^24,26,49^ At one end of the spacer, both strands extend as a 12nt cholesterol-modified ssDNA segment, which enable stable insertion of the amphiphilic constructs into the membranes.^47^ At the opposite end of the tendril, ssDNA sticky ends (or prongs) are present. By design, the prongs of *T*_L_ and *T*_U_ are complementary (domains *θ* and *δ* on *T*_U_, and θ* and δ* on *T*_L_), constituting a double-toehold system that, upon L–U binding, triggers a 4-way branch migration involving stem domains *ζ* and ζ*, leading to a zipping action.^50–53^ After the branch migration is completed, zipping progresses all the way to the cholesterol attachment points, thanks to the cross-complementarity of the cholesterolised ssDNA domains (*α*/α* and *β*/β*) between the two tendrils, bringing the membranes within molecular proximity and promoting fusion. As it involves hybridisation of previously unpaired domains rather than branch migration, this last step provides a strong thermodynamic drive for completing the zipping. Note that while *α*/α* and *β*/β* domains may, in principle, initiate branch migration events similarly to the sticky ends, this is expected to be unlikely due to their comparatively lower accessibility.

Comparison between the performance of tendrils and simple ssDNA zippers is summarised in Fig. 1c and d, highlighting a substantial advantage of the former over the latter (see Fig. S1 and S2, ESI† for additional experiments). Tendrils lacking one of the cholesterol molecules, but retaining the double-stranded structure, show intermediate performance between the flexible zippers and the complete, two-anchor tendrils, demonstrating the beneficial role of both the dsDNA spacer and the double-cholesterol moiety.^30^

**Fig. 2:**
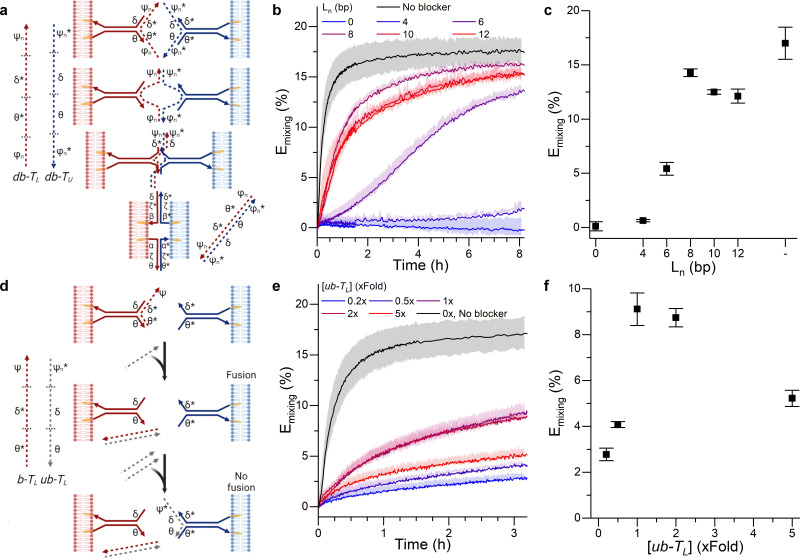
DNA circuits can be used to control membrane fusion. (a) The addition of blocking strands (dashed) to each of the tendrils delays the interaction between them, as tendril zipping requires the prior displacement of the blocking motifs. (b) Time evolution and (c) values recorded at time = 3 hours of the FRET efficiency *E*_mixing_ for increasing lengths (*L*_n_) of the blocker overhangs. (d) Blocking a single tendril enables the creation of a band-pass filter. The addition of a trigger strand (*ub* − *T*_L_) which preferentially binds the blocking motif *b* − *T*_L_ frees the blocked tendril, thus enabling membrane fusion. However, if excess *ub* − *T*_L_ is added, it will bind the non-blocked tendril, resulting in an overall reduction of the fusion efficiency. (e) Kinetic and (f) values at time = 3 hours for *E*_mixing_ at increasing concentrations of *ub* − *T*_L_, demonstrating optimal fusion efficiencies at intermediate values and thus the sought band-pass filtering effect. See Tables S1 and S2 (ESI†) for ssDNA sequences and strands used for each DNA construct.

The tendril architecture enables facile control over fusion efficiency exploiting non-cholesterolised blocker strands, which hybridise to the prongs, regulating their availability, as summarised in Fig. 2a and d. The modification of blocker strands enables the triggering and temporal control of the fusion process without the need for changing the fusogenic domain, contrary to other approaches that primarily rely on sequential fusion steps of uniquely labelled liposome populations, thus requiring multiple cholesterol-functionalised DNA components.^27,41,54^ As a proof of concept, we show how blocker strands can be used to suppress (Fig. S3, ESI†) or modulate the fusion kinetics (Fig. 2a–c), and engineer a bandpass-like fusion behaviour (Fig. 2d–f).

In order to modulate the fusion kinetics, tendrils were pre-hybridised to d*b* − *T*_L_ and d*b* − *T*_U_ blocking strands, complementary to the prongs of *T*_L_ and *T*_U_, respectively, and thus inhibiting fusion. The blocking strand d*b* − *T*_L_, however, features ssDNA overhangs of length *L*_n_, *φ*_n_ and *ψ*_n_, complementary to 
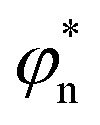
 and 
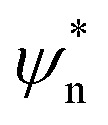
 on d*b* − *T*_U_. These overhangs enable recognition between tendrils by exposing the protected prongs following a toehold-mediated strand displacement process (Fig. 2a). Therefore, by controlling the toehold length *L*_n_ it is possible to modulate the strand displacement kinetics between the blocking strands,^51^ ultimately controlling the rate of membrane fusion (Fig. 2b and c). In turn, if a single tendril (*e.g. T*_L_) is blocked (by blocker *b* − *T*_L_), fusion efficiency is expected to proceed upon the addition of the corresponding unblocker strand (*ub* − *T*_L_), as depicted in Fig. 2d. However, we observed that increasing *ub* − *T*_L_ concentration only led to an increase in fusion rate until the concentration of *T*_L_ was matched, decreasing once more at higher *ub* − *T*_L_ concentrations (Fig. 2e and f). This behaviour can be rationalised by considering the relative affinities of *ub* − *T*_L_ for *b* − *T*_L_ and *T*_U_. *ub* − *T*_L_ is fully complementary to *b* − *T*_L_ and therefore, they will dimerise preferentially, leading to the unblocking of *T*_L_ and enabling membrane fusion. When the concentration of *ub* − *T*_L_ goes beyond that of *b* − *T*_L_, the excess unblocker strand can bind *T*_U_ prongs, thus inhibiting fusion despite *T*_L_ being unblocked. Similar “band-pass” filter behaviour has been demonstrated in genetic circuits, where it constituted the basis for spatial patterning.^55^

### 2.3 Effect of lipid type on DNA-mediated membrane fusion

Having presented an efficient and controllable fusogenic DNA design, we proceed to explore the influence of lipid composition on fusion efficiency. For these experiments, the composition of L liposomes was systematically varied, while using the gold standard composition (50/25/25 DOPC/DOPE/Chol) for U vesicles throughout all the experiments.

#### 2.3.1 Increased DOPE and cholesterol concentration improves lipid mixing

We first considered binary DOPE/DOPC mixtures for the L vesicles, and systematically explored the effect of changing the molar fraction of the Type II lipid (DOPE), *χ*_DOPE_. As shown in Fig. S4a and d (ESI†), no significant lipid mixing was measured up to *χ*_DOPE_ = 0.1, which then increased roughly linearly up to *E*_mixing_ ∼ 25% at *χ*_DOPE_ = 0.75. This observation is consistent with previous reports suggesting the emergence of the hexagonal *H*_II_ phase at *χ*_DOPE_ > 0.5 under excess hydration conditions,^56^ which we argue may play a role in stabilising the fusion stalk. We then evaluated the extent of lipid mixing at the same relative DOPE/DOPC ratio in liposomes containing 25% mol cholesterol. As seen in Fig. S4b and e (ESI†), lipid mixing efficiency increased regardless of the DOPE/DOPC ratio, compared to cholesterol-free bilayers. The fusogenic effect of cholesterol could be two-fold: on the one hand, cholesterol may increase the lipid packing stress, leading to the formation of membrane defects which promote fusion, while on the other hand it induces dehydration of the membrane interface, thus facilitating the approach between the fusogenic bilayers.^57–59^ Consistently, further increasing cholesterol content up to 50% mol caused a further increase in fusion efficiency, as displayed in Fig. S4c and f (ESI†).

Overall these results highlight the importance of a rational choice of liposome composition when designing fusogenic vesicles. For instance, by combining the highly fusogenic DOPE lipid with cholesterol the attractive interaction between PE headgroups and water^60^ is counteracted by cholesterol-induced membrane dehydration, thus maximising the fusogenic potential of the membrane while retaining a stable membrane structure.

Having assessed the effect of fusogenic lipid (DOPE) and cholesterol content, we move on to systematically exploring how changing the identity of the structural (lamellar forming, *e.g.* DOPC) or the fusogenic (*H*_II_ promoting, *e.g.* DOPE) lipids affected the extent of lipid mixing.

#### 2.3.2 Membrane-curvature stress is required for successful fusion

We explored the effect of replacing DOPE for other fusogenic lipid types in L liposomes using 50/25/25 DOPC/X/Chol compositions where X indicates the fusogenic component chosen: POPE, POPA, oleic acid (OA) or DOPC. These lipids were selected according to their spontaneous curvature and charge. POPE is analogous to DOPE except for having only one unsaturated chain (as opposed to DOPE, where both chains contain a double bond), and therefore it displays a lower Type II character (*e.g.* lower spontaneous curvature *J*_0_). The hydrocarbon chains of POPA are analogous to that of POPE, however, its headgroup is smaller (thus promoting a higher membrane curvature) but negatively charged. OA acid consists of a single, unsaturated chain and does also promote the formation of highly-curved membranes.^61^ DOPC was selected as zero-curvature control lipid.

**Fig. 3:**
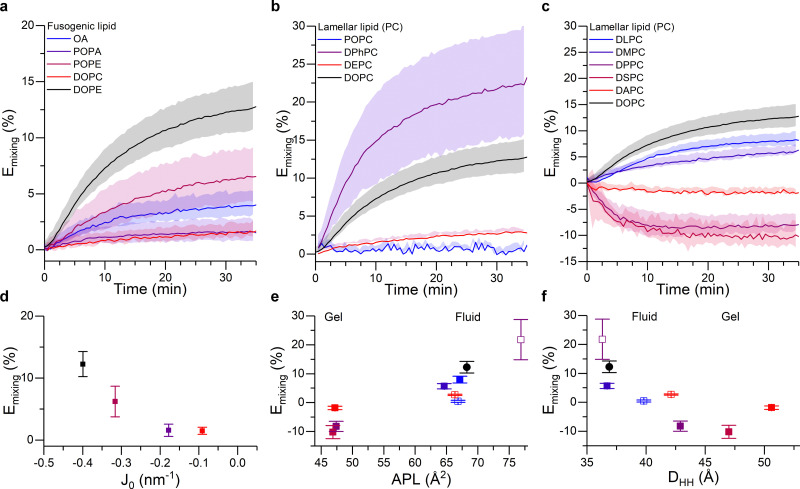
Membrane composition and structural properties modulate fusion efficiency. (a–c) Kinetic traces showing the FRET-based proxy for lipid mixing efficiency, *E*_mixing_, for: (a) 50/25/25 DOPC/X/Chol liposomes, where the fusogenic lipid (X) is systematically varied (see legend and note that DOPC was included as non-conical control); (b) 50/25/25 X/DOPE/Chol liposomes, where the unsaturation type of the lamellar lipid is varied (see legend); (c) 50/25/25 X/DOPE/Chol liposomes, where the length of the saturated acyl chains is systematically increased (see legend). Chain lengths are 12, 14, 16, 18 and 20 for DLPC, DMPC, DPPC, DSPC and DAPC, respectively. DOPC was included as a control. (d–f) Mixing efficiency 35 minutes after the addition of U vesicles, shown as a function of (d) spontaneous curvature, *J*_0_, of the fusogenic lipid (note *J*_0_ values for OA were not available in the literature), (e) area per lipid and (f) membrane thickness of bilayers formed by the corresponding lamellar lipid. Color coding in panels (d–f) matches that in (a–c). For (e and f) empty symbols correspond to the lipids used in (b) and filled dots represent the traces in (c). See ESI† for details on the membrane biophysical properties of the membranes.

As shown in Fig. 3a, DOPE remained the lipid type with the highest fusogenic potential. This can be justified on the grounds of the induced monolayer curvature, noting a clear correlation between fusion efficiency and spontaneous curvature *J*_0_ of the “conical” component (Fig. 3d). Moreover, our data suggests a minimum spontaneous curvature is required for the fusion process to initiate – in this experiment falling between the *J*_0_ values of POPA and POPE – likely because such geometry is required to stabilise the intermediate fusion stalk.

#### 2.3.3 Structural lipid type modulates fusion efficiency and the morphology of the fusion product

Next, we investigated the effect of modifying the length and degree of unsaturation of the structural, lamellar-forming PC lipids in X/DOPE/Chol 50/25/25 L liposomes. We tested fully saturated PC compositions with a hydrocarbon length ranging from 12 to 20 carbon atoms (12 – DLPC, 14 –DMPC, 16 – DPPC, 18 – DSPC and 20 – DAPC), together with the lipids POPC – containing a single double bond, DEPC – contaning two double bonds in *trans* configuration, and DPhPC – having fully saturated, but methylated chains (full list of names given in the ESI†). The long chain lengths of DPPC, DSPC and DAPC increase lipid packing, driving the membrane towards a gel phase.^62^ It is expected that these long-tail lipids will cluster together and phase separate from the more fluid (DOPE-rich) regions, while the rest of the selected lipid molecules will keep the bilayer in an homogeneous, fluid state.^63^ Therefore, the presence of gel-forming PC lipids could trigger the formation of membrane domains, and in turn this may facilitate membrane fusion.^27^ In addition, by varying the nature of the hydrocarbon region we controlled the elastic properties^64^ of the fusogenic liposomes, which in turn would influence the fusion efficiency.^65^

Fig. 3b and c shows the extent of lipid mixing for the aforementioned L vesicle compositions. The highest lipid mixing efficiency was observed when DPhPC was used as the structural lipid, in agreement with existing work suggesting the higher membrane fluidity^66^ and higher tendency to form rhombohedral phases of DPhPC,^33,44^ which we argue may facilitate the fusion process. All the other lipid types forming fluid phase bilayers showed a decreasing fusion efficiency in the order: DPhPC > DOPC > DLPC > DMPC > DEPC > POPC. Remarkably, this relationship correlated well with the area per lipid (APL) and thickness (*D*_HH_) of pure bilayers formed by these species, as evidenced in Fig. 3e and f. Such relation can be justified in terms of lipid geometry and membrane remodelling. In the first case, because all the tested lipids have the same PC headgroup, changes in the APL will predominantly be due to the space occupied by the acyl chain and, therefore, lipids with higher APL will have a stronger Type II character, which would facilitate fusion. In addition we observed a negative correlation between lipid fusion efficiency and membrane thickness, arguably because thicker membranes generally have higher bending modulus, therefore making membrane bending towards fusion intermediates more energetically costly. Overall, these results further highlight the importance of molecular organisation in the membranes, and therefore their mechanical properties, on the efficiency of the fusion process.

Surprisingly, we observed an increase in FRET efficiency (*E*_mixing_ < 0) after fusion when DPPC, DSPC or DAPC lipids were used as the structural component of the L vesicles, which would paradoxically indicate a “negative” lipid mixing efficiency. Successful fusion was independently confirmed through the Tb/DPA content mixing assay (Fig. S5, ESI†), suggesting that the unexpected trend in FRET efficiency resulted from the fusion process.

The three structural lipids leading to the anomalous result are known to promote the emergence of highly ordered gel and liquid ordered lipid domains. We can therefore speculate that the fusion of these vesicles with DOPC/DOPE/Chol U liposomes could lead to membrane remodelling and phase separation, which may in turn be the cause of the observed FRET response, *e.g.* by leading to an increase in the local concentration of the fluorescently labelled species and a consequent reduction in their separation and thus an increase in FRET efficiency.

**Fig. 4:**
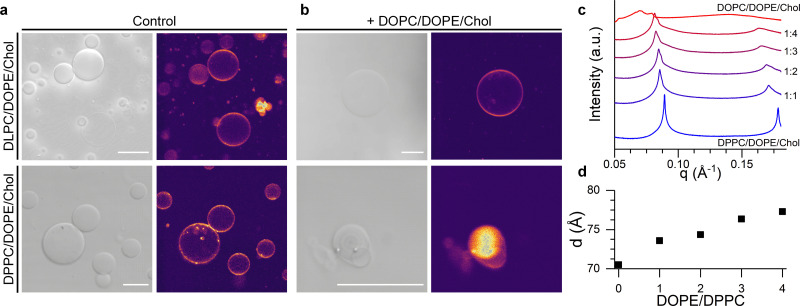
Structural, lamellar lipids determine the morphology and lateral organisation of the fusion product. (a) Brightfield and confocal fluorescence microsopy (CLSM) images of DLPC/DOPE/Chol and DPPC/DOPE/Chol GUVs. (b) Brightfield and CLSM of the GUVs electroformed using the expected composition resulting from fusion with DOPC/DOPE/Chol liposomes at a 1 : 4 ratio. Scalebar: 20 μm (c) SAXS traces showing phase-separation induced by the addition of DOPC/DOPE/Chol mixtures to DPPC/DOPE/Chol membranes. (d) Change in the lamellar repeat distance *d* with increasing DOPE/DPPC ratio. See Table S4, ESI,† for lipid compositions used in microscopy and SAXS experiments.

In order to test this hypothesis, we formed giant unilamellar vesicles (GUVs) corresponding to the L-only liposome composition and to the lipid mixture expected after fusion with the U vesicles. Confocal images in Fig. 4a and b and Fig. S6 (ESI†) show that mixing of DPPC/DOPE/Chol with DOPC/DOPE/Chol compositions lead to smaller GUVs displaying nested, multilamellar architectures whereas, when DLPC/DOPE/Chol was used as the starting composition, membrane morphology remained unchanged. However, the homogeneous fluorescence across all the tested GUV compositions indicated a lack of macroscopic phase separation, in agreement with previous reports.^67^

In order to gain further evidence of a structural change in DPPC/DOPE/Chol liposomes upon fusion with DOPC/DOPE/Chol vesicles, we performed small angle X-ray scattering (SAXS) measurements on samples with increasing DOPC/DPPC ratio, hence mimicking the presumed evolution in membrane composition during the fusion process. As shown in Fig. 4c and d, data from pure DPPC/DOPE/Chol membranes shows a set of individual peaks, corresponding to a lamellar diffraction pattern with a *d*-spacing of ∼70.5 Å. As the relative amount of DOPC/DOPE/Chol increases, the SAXS peaks become broader and a shoulder starts to appear at higher wave vectors, suggesting an heterogeneous lateral membrane organisation typical of domain formation, in agreement with the increase in *E*_mixing_ observed from our fluorescence studies. Concurrently, the increasing fraction of DOPC/DOPE/Chol caused an increase in the interlamellar repeat *d*-spacing (Fig. 4d) from 70.5 Å to 77.3 Å, which is compatible with an increased membrane curvature stress, as previously reported by Tyler *et al.*^61^

Given the evidence of a structural change in the bilayers induced by membrane fusion, we hypothesise that the process could be exploited to actively control the bilayer lateral organisation and stress, thus modulating its mechanical behaviour.

#### 2.3.4 DNA mediated fusion controls membrane mechanics

In order to test this hypothesis, we monitored the changes in membrane fluidity of L liposomes after fusion using Laurdan (6-dodecanoyl-2-dimethylaminonaphthalene) fluorescence measurements. Laurdan is a solvatochromic fluorescent probe, which spontaneously partitions in the bilayers and reports on membrane polarity/hydration – a proxy for membrane fluidity – through a shift in the emission spectrum. In polar, more fluid membranes Laurdan emission is red-shifted, while the spectra shift to shorter wavelengths in a highly ordered environment. The ratio between the blue and red shifted emission (the so called Laurdan General Polarisation – GP, see ESI†) is indicative of the degree of lipid packing, where high GP values correspond to a higher membrane order and lower fluidity.^68^

**Fig. 5:**
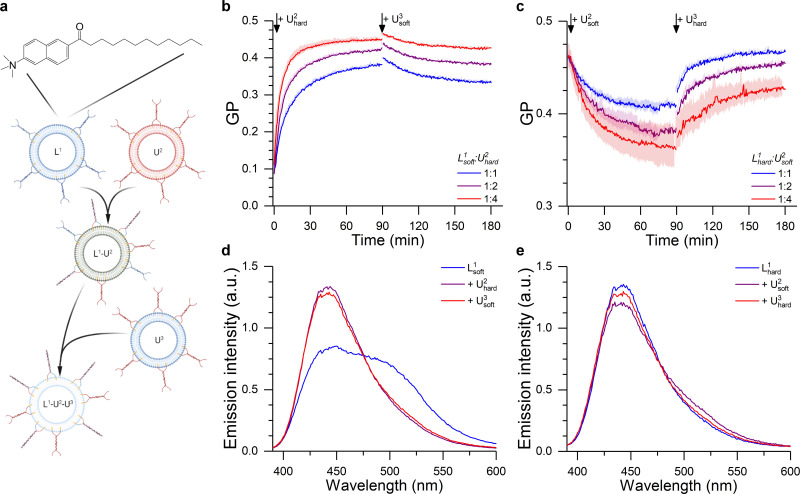
DNA-Mediated fusion as tool to program the membrane mechanical properties. (a) The fusion between a Laurdan-labelled vesicle (L^1^) with an unlabelled liposome (U^2^) of a different composition alters the bilayer structure of L^1^, as evidenced by the change in Laurdan GP (see ESI†). The fusion product L^1^–U^2^ may then able to further fuse with another set of unlabelled liposomes (U^3^), resulting in a further change in membrane properties. (b) Kinetic traces showing the hardening of 50/25/25 DOPC/DOPE/Chol LUVs (L^1^_soft_) after the addition of 50/25/25 DPPC/DOPE/Chol liposomes (U^2^_hard_), as suggested by the change in Laurdan GP. This effect was partly reverted through the addition of 50/25/25 DOPC/DOPE/Chol LUVs (U^3^_soft_) at time = 90 min. (c) Laurdan GP kinetic traces showing the softening of Laurdan labelled 50/25/25 DPPC/DOPE/Chol LUVs (L^1^_hard_) after the addition of 50/25/25 DOPC/DOPE/Chol (U^2^_soft_) liposomes. This effect was reverted through the addition of 50/25/25 DPPC/DOPE/Chol LUVs (U^3^_hard_) at time = 90 min. L^1^/U^2^ ratio was varied as indicated by the legend, while L^1^/U^3^ was 1 : 4 in all cases. (d and e) Laurdan emission spectra before and after the 1st and 2nd fusion processes corresponding to the traces shown in b,c) for a 1 : 4 L^1^ to U^2^ ratio.

Initially, we considered less packed, “softer” DOPC/DOPE/Chol liposomes displaying a fluid L_α_ phase (Fig. S7, ESI†) (here dubbed L^1^_soft_ and functionalised with *T*_L_ tendrils) and tested whether their lipid order and mechanical properties could be altered through fusion with “harder” DPPC/DOPE/Chol vesicles in a liquid ordered L_o_ phase (dubbed U^2^_hard_ and functionalsied with *T*_U_ tendrils). These changes where monitored by labelling L^1^_soft_ with Laurdan (Fig. 5a). Importantly, the tight anchoring of Laurdan to bilayers, and its very low water solubility, will prevent it from spontaneously detaching from L^1^_soft_ liposomes and redistributing to other populations.^69,70^ Therefore, we expect the changes in Laurdan GP to be caused by the change in lipid composition of the L^1^_soft_ liposomes as a result of fusion. As summarised in Fig. 5b (see associated Laurdan spectra in Fig. 5d), the initial GP value of L^1^_soft_ liposomes was measured as ∼0.1, in agreement with previous reports.^71^ The GP value increased upon fusion with U^2^_hard_ liposomes, with larger shift being observed with increasing U^2^_hard_/L^1^_soft_ ratios, as expected. The highest recorded GP of ∼0.45 is similar to that of pure DPPC/DOPE/Chol membranes (Fig. 5b) suggesting a high fusion efficiency.

We then sought to verify whether the hardening process could be reversed through fusion with soft DOPC/DOPE/Chol membranes, in the form of U^3^_soft_ liposomes labelled with *T*_U_ tendrils. However, when U^3^_soft_ liposomes were added to the L^1^_soft_–U^2^_hard_ fusion product, the GP values did not return to the original levels (Fig. 5d) and, in fact, the amount of GP recovery was inversely correlated to the amount of U^2^_hard_ present in the first fusion step. Arguably, as the amount of fused U^2^_hard_ increases, the membrane becomes less fusogenic and, therefore, the second fusion round becomes less efficient.

In agreement with this observation, when DOPC/DOPE/Chol (U^2^_soft_) LUVs were made to fuse with Laurdan-labelled DPPC/DOPE/Chol (L^2^_hard_) liposomes, the decrease in GP value did not reach that of pure U^2^_soft_ (Fig. 5c and d). In this case, the lower fusogenic potential of the L^1^_hard_ membranes could be preventing the efficient incorporation of lipids and the fluidizing effect of U^2^_soft_ liposomes, justifying the smaller change in GP. However, a full recovery of the GP towards the initial value is observed after further addition of DPPC/DOPE/Chol (U^3^_hard_) vesicles, presumably facilitated by the increase in fusogenicity of the Laurdan-labelled species induced by the initial fusion round.

We note that, in these experiments, the depletion of unpaired tendrils following the first fusion step may play a role in limiting the efficiency of the second step. In addition, we note that composition, and thus mechanical properties, are likely to be quite variable across the population of fusion products, owing to the stochastic nature of the fusion process. To mitigate against this polydispersity, one may rationally design a negative feedback loop whereby alterations in composition following fusion hinder further fusion events, so to select products of defined composition. The aforementioned depletion of available fusogenic nanostructure following initial fusion events may also help limiting variability.

Overall, results discussed in this section demonstrate how fusion can be used to control the mechanical properties of membranes and how, in turn, these changes influence the fusogenic potential of the products membranes giving rise to positive or negative feedbacks. These behaviours are a simple example of the emerging complexity that can be accessed when utilising lipid composition to program fusogenicity.

## 3 Conclusions

Engineering the fusion between bilayer membranes is likely to become an increasingly important bio-technological task, as we grow more reliant on lipid-based nano-medical vectors as a means of delivering vaccines and other therapeutics, and as we make progress with replicating complex life-like behaviours in synthetic cellular systems. There is therefore a pressing need for expanding the array of available tools to program membrane fusion and, importantly, consolidating and synergising independent methodologies. In this work, we tackle this urgent challenge from two different angles, namely the design of more efficient and easily programmable fusogenic, DNA-based, nanostructures and the systematic characterisation of the effects of changing the bio-physical features of the membranes.

First, we introduce DNA tendrils, efficient fusogenic constructs that can be easily coupled with soluble DNA motifs, unlocking a route for easily and cheaply modulating fusion efficiency without the need to redesign the main components. Second, we report on a complete characterisation of the effect of varying the proportions and the chemical identity of the molecular components in the fusogenic bilayers, namely the non-fusogenic lamellar lipids, the fusogenic conical lipids and sterols. We highlight general trends that link the fusion efficiency to the intrinsic curvature of the conical lipids, and the head-group area and bilayer thickness of the lamellar components. The observed trends are rationalised based on the stabilising or de-stabilising effects that these biophysical parameters have on the transient membrane morphologies thought to appear in the fusion pathways, and may provide guidelines for a more informed design of fusogenic compositions. We also observe unexpected behaviours in the presence of gel-forming saturated lamellar components, which we ascribe to phase separation occurring upon fusion with liquid membranes. Finally, we demonstrate how fusion between vesicles with distinct mechanical properties can be used to program changes in the fluidity of the fusion products, and how the latter, in turn, has a feedback effect on the fusion efficiency.

Taken together, our nanodevice designs and observations strengthen and expand the toolkit for programming lipid membrane fusion trough orthogonal approaches, meeting the demand for versatility emerging from the diverse applications that require control over this ubiquitous biophysical process.

## Conflicts of interest

The authors declare no conflicts of interest.

## Supplementary Material

SM-018-D2SM00863G-s001
