# Supplementary material for: Modulating membrane fusion through the design of fusogenic DNA circuits and bilayer composition
Source: Soft Matter. 2022 Aug 17;18(37):7035–44. doi: 10.1039/d2sm00863g (PMC9516350; doi:10.1039/d2sm00863g)
Supplement: SM-018-D2SM00863G-s001 [file SM-018-D2SM00863G-s001.pdf]

Electronic Supplementary Material (ESI) for Soft Matter.
This journal is © The Royal Society of Chemistry 2022

ELECTRONIC SUPPLEMENTARY INFORMATION

Modulating membrane fusion through the design of fusogenic DNA circuits and bilayer composition

Miguel Paez-Perez,[a,b] I Alasdair Russell[c], Pietro Cicuta[d] and Lorenzo Di Michele*[a,b,d]

a. *Molecular Sciences Research Hub, Department of Chemistry, Imperial College London, Wood Lane, London, W12 0BZ, UK*
b. *fabriCELL, Imperial College London, Wood Lane, London, W12 0BZ, UK*
c. *Cancer Research UK Cambridge Institute, University of Cambridge, Cambridge CB2 0RE, UK*
d. *Biological and Soft Systems, Cavendish Laboratory, University of Cambridge, Cambridge CB3 0HE, UK*

Experimental methods:

Materials:
Lipids solutions in $CHCl_3$ of 1,2-dioleoyl-sn-glycero-3-phosphocholine (DOPC), 1-palmitoyl-2-oleoyl-glycero-3-phosphocholine (POPC), 1,2-diphytanoyl-sn-glycero-3-phosphocholine (DPhPC) 1,2-dielaidoyl-sn-glycero-3-phosphocholine (DEPC), 1,2-dilauroyl-sn-glycero-3-phosphocholine (DLPC), 1,2-dimyristoyl-sn-glycero-3-phosphocholine (DMPC), 1,2-dipalmitoyl-sn-glycero-3-phosphocholine (DPPC), 1,2-distearoyl-sn-glycero-3-phosphocholine (DSPC), 1,2-diarachidoyl-sn-glycero-3-phosphocholine (DAPC), 1,2-dioleoyl-sn-glycero-3-phosphoethanolamine (DOPE) 1-palmitoyl-2-oleoyl-sn-glycero-3-phosphoethanolamine (POPE) and 1-palmitoyl-2-oleoyl-sn-glycero-3-phosphate (sodium salt) (POPA) were purchased from Avanti Polar Lipids®. Laurdan was obtained from ThermoFisher, and dissolved in CHCl3 to prepare a 300µM stock solution.
RP-HPLC purified cholesterol-modified DNA (Table S1) and unmodified DNA oligonucleotides were provided by Eurogentec and IDT, respectively, and resuspended in Tris EDTA (TE) buffer (pH 8) to a 100 µM final concentration. All other reagents were purchased from Sigma Aldrich® or VWR and used without further purification.

Tendril annealing:
The fusogenic DNA nanostructures (*tendrils*) were prepared by mixing equimolar amounts of the corresponding oligonucleotides (Table S2) to a final concentration of 10µM (*i.e.* individual oligo concentration) in 100mM NaCl TE (pH 7.4). The mixture was then annealed by heating to 95°C and then cooling at a rate of -0.5°C/min to a temperature of 15°C.

Large Unilamellar Vesicle (LUV) formation:
Lipids dissolved in $CHCl_3$ were mixed to the appropriate molar ratio (Table S3) and, for labelled *L* liposomes, either Rhodamine-PE and NBD-PE (1%mol each) or Laurdan (1%mol) were added. The organic solvent was then removed using a rotatory evaporator to create a lipid film, which was then dried overnight under vacuum. Afterwards, it was hydrated by adding 100mM NaCl TE buffer (pH 7.4) to a final concentration of 2mg/mL total lipid and vortexed until the solution turned cloudy and the lipid film could no longer be seen. We notice resuspension of DPhPC/DOPE/Chol and DOPC/OA/Chol films required one freeze-thaw cycle. This suspension was than extruded 15 times (above the transition temperature of the lipid with the highest T_m) through a 100nm polycarbonate filter (Avanti Polar Lipids®).

In the case of content mixing assay, vesicles were resuspended in either 15mM $TbCl_3$, 150mM sodium citrate, 5mM NaCl, 10mM Tris or 150mM DPA, 25mM NaCl, 10mM Tris buffer, for *L* and *U* liposomes, respectively. The pH of both buffers was adjusted to ~7, and freeze-thawing was performed to improve the encapsulation efficiency. After extrusion, the loaded LUVs were purified using a NAP-10 size exclusion column.

LUV functionalization with DNA tendrils:
Vesicle decoration was performed by mixing labelled (*L*) (5µL, 2mg/mL lipid) and unlabelled (*U*) 15µL, 2mg/mL) LUVs with the tendrils (T_L: 7.62µL, 10µM and T_U: 5.49µL, 10µM respectively), which resulted in an estimated tendril:vesicle ratio of 200:1 and 50:1 respectively. These solutions were further diluted in 100mM NaCl TE (pH 7.4) buffer to achieve a final lipid concentration of 0.5mg/mL, and the mixture was incubated overnight at room temperature before use.

Fluorescence spectra and kinetics measurements:
In a NUNC-96 well plate, 100µL 100mM NaCl TE (pH 7.4) and 4µL of *L* vesicles (0.5mg/mL) were mixed with 16µL of *U* liposomes (0.5mg/mL). The fusion kinetics were then recorded using a BMG CLARIOstar Plus plate reader at 530±10nm and 590±10nm emission after 465±10nm excitation (NBD/Rh FRET) or 435±10nm and 490±10nm emission under 360±10nm nm excitation (Laurdan). In the case of FRET experiments, 4µL of 2% Triton-X detergent were added and the measured NBD and Rh intensities were used as the *infinite-dilution* values.

The extent of lipid mixing (E_{mixing}) was quantified as:

$$E_{mixing} = \frac{\varepsilon(t) - \varepsilon_0}{\varepsilon_{TX} - \varepsilon_0} \qquad (1)$$

where $\varepsilon(t)$ is the FRET efficiency at time t, ε_0 is the FRET efficiency at t=0 and ε_{TX} is the FRET efficiency after LUV disruption using detergent Triton-X. In all cases, the FRET efficiency ε was approximated from the donor (I_D) and acceptor (I_A) intensities as:

$$\varepsilon = \frac{I_A}{I_D + I_A} \qquad (2)$$

We note E_{mixing} is not an actual measure of the fusion progress. First, the described FRET assay is sensitive to lipid mixing between the leaflets of different vesicles, but this may not necessarily lead to full fusion (*i.e.* content mixing). Secondly, previous work has highlighted the effect of membrane structure and detergent use in the extent of fluorophore dequenching upon solubilization of the membrane[1] and, lastly, FRET assays are dependent on the distance between the fluorescent dyes, which in turn will be dictated by the degree of lipid packing and/or the area per lipid. Therefore, proper quantification of lipid mixing would require individual calibrations for each individual *L/U* combination. However, such approach was deemed unfeasible due to the large number of lipid compositions used in this work.

When Laurdan was used, the lipid ordering of the membrane was evaluated through the General Polarization (GP) function defined as:

$$GP = \frac{I_{435\pm2} - I_{490\pm2}}{I_{435\pm2} + I_{490\pm2}} \qquad (3)$$

Kinetic and fixed-time data is presented as mean ± s.d. for $n \geq 3$.

For the content mixing assay, the samples were placed in a 50µL low-volume quartz cuvette instead, and $Tb(DPA)_3$ fluorescence intensity was measured using a Horiba Yvon Fluoromax 4 fluorimeter under 260±3nm excitation and 544±3nm emission (n=1).

<u>Giant Unilamellar Vesicle (GUV) formation:</u>
30µL of a 1mg/mL lipid solution with the indicated composition (Table S4) was spread onto an ITO slide. After drying for >1h under vacuum, a PDMS spacer was pressed onto the slide, the chamber was filled with a 400mM sucrose solution and was then closed with a second ITO slide. The electroformation protocol consisted of the application of 1Vpp@10Hz electric field for 90 min, followed by a 30 min detachment phase at 1Vpp@2Hz. The resulting GUVs were recovered by gently tilting the glass slide (to avoid GUV bursting due to shear exerted during pipette aspiration).

GUVs were then diluted ten-fold before imaging (using a custom-made PDMS chamber on top of a BSA-coated glass slide).

<u>Small angle X-Ray Scattering experiments:</u>
Dry samples of a given lipid mixture (20mg total mass) were hydrated with DI water to 70% and subjected to 15 freeze-thaw cycles to ensure a proper lipid mixing. The sample was then loaded into a 2mm diameter polymer capillary tube and sealed with a rubber stopper. SAXS measurements were performed at beamline I22 (Diamond Light Source, UK).[2] The diffraction profiles were obtained from the radial integration of the 2D SAXS patterns, and peak position and width was determined by fitting them to pseudo-Voigt functions using a custom-built MATLAB® script.

Note on the lipid parameters presented in Fig. 3d-f:

The area per lipid and membrane thickness values were obtained from Kučerka et al.[3,4] and/or from Sun et al.[5] DEPC parameters were derived from our own measurements. The values for lipid curvature (DOPC, POPE, DOPE) were extracted from Kollmitzer et al.[6] In the case of POPA, the spontaneous curvature was approximated from that of DOPA[7], and then corrected as:

$$J_0^{POPA} = J_0^{DOPA} \frac{J_0^{POPE}}{J_0^{DOPE}} \qquad (4)$$

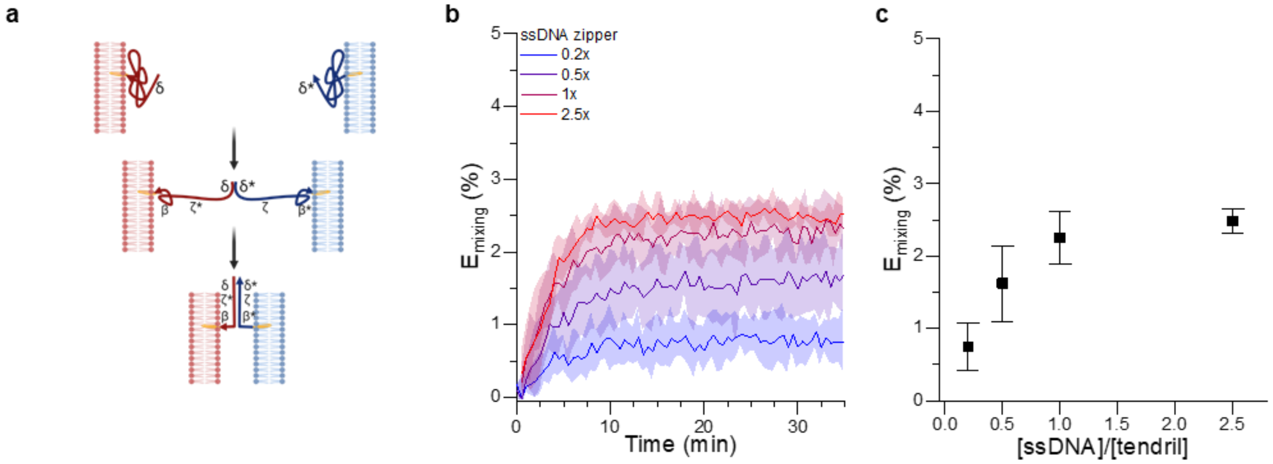

Fig. S1 | Fusion-induced lipid mixing efficiency using a single-strand DNA zippers. a) Plausible pathway leading to zipping of ssDNA constructs, initially coiled up. **b)** Kinetic traces showing mixing at a range of zipper concentrations expressed as fractions of the concentration of tendrils used throughout this work (1x, corresponding to 200 fusogenic DNA structures per *L* LUV and 50 fusogenic DNA structures per *U* LUV). **c)** E_{mixing} after 35 minutes from the beginning to the experiment as a function of zipper concentration. 1x represents the standard coverage used throughout this work. Both *L* and *U* LUVs had a 50/25/25 DOPC/DOPE/Chol composition.

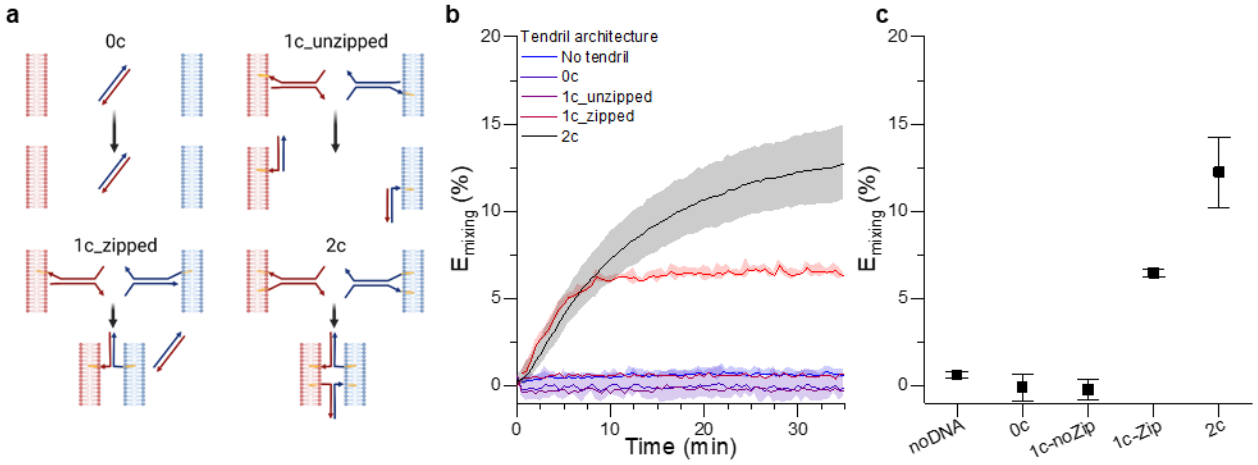

Fig. S2 | Effect of tendril architecture on lipid mixing efficiency. a) DNA construct binding and (possible) consequent membrane fusion for various control design used in this work. Design 2c is the "standard" tendril, with two cholesterol anchors on each construct. Design1c_zipped has a single cholesterol moiety on each tendril, but two cholesterolised ssDNA in the two tendrils are complementary, leading to membrane-membrane binding and zipping action. Tendrils with 1c_unzipped design have a single cholesterol on non-complementary ssDNA elements, so no zipping and permanent membrane binding is possible. Design 0c lacks cholesterol moieties and does not bind to the bilayers. **b)** Kinetic traces showing E_{mixing} for different tendril designs. **c)** E_{mixing} at 35 minutes. Both *L* and *U* LUVs had a 50/25/25 DOPC/DOPE/Chol composition.

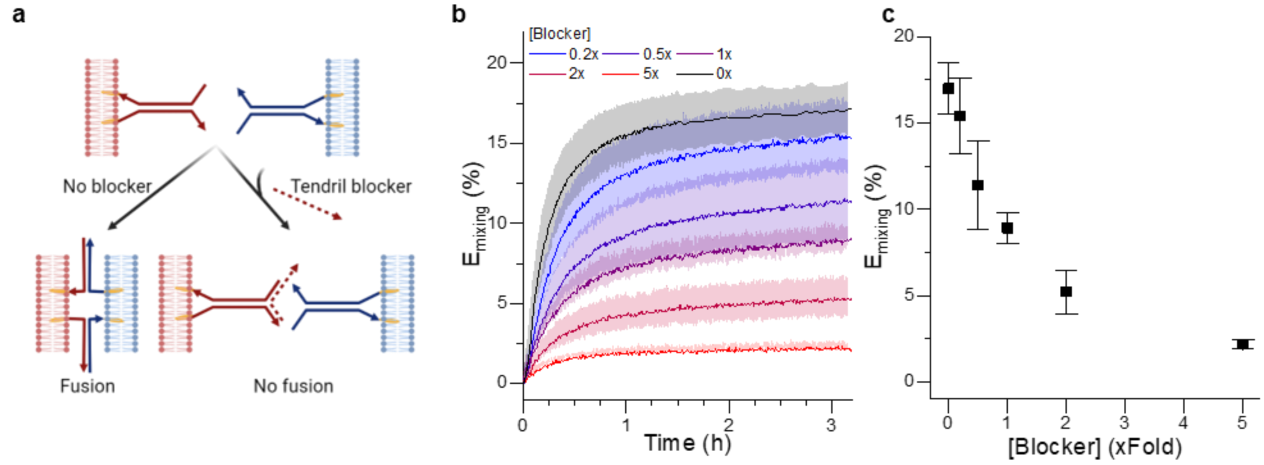

Fig. S3 | Effect of blocker concentration on lipid mixing efficiency. a) Blocker addition hinders the interaction between sticky ends on the tendrils, this preventing fusion. **b)** Kinetic traces showing E_{mixing} for increasing blocker concentration. **c)** E_{mixing} at 3h as a function of blocker concentration. Both donor and acceptor LUVs had a 50/25/25 DOPC/DOPE/Chol composition.

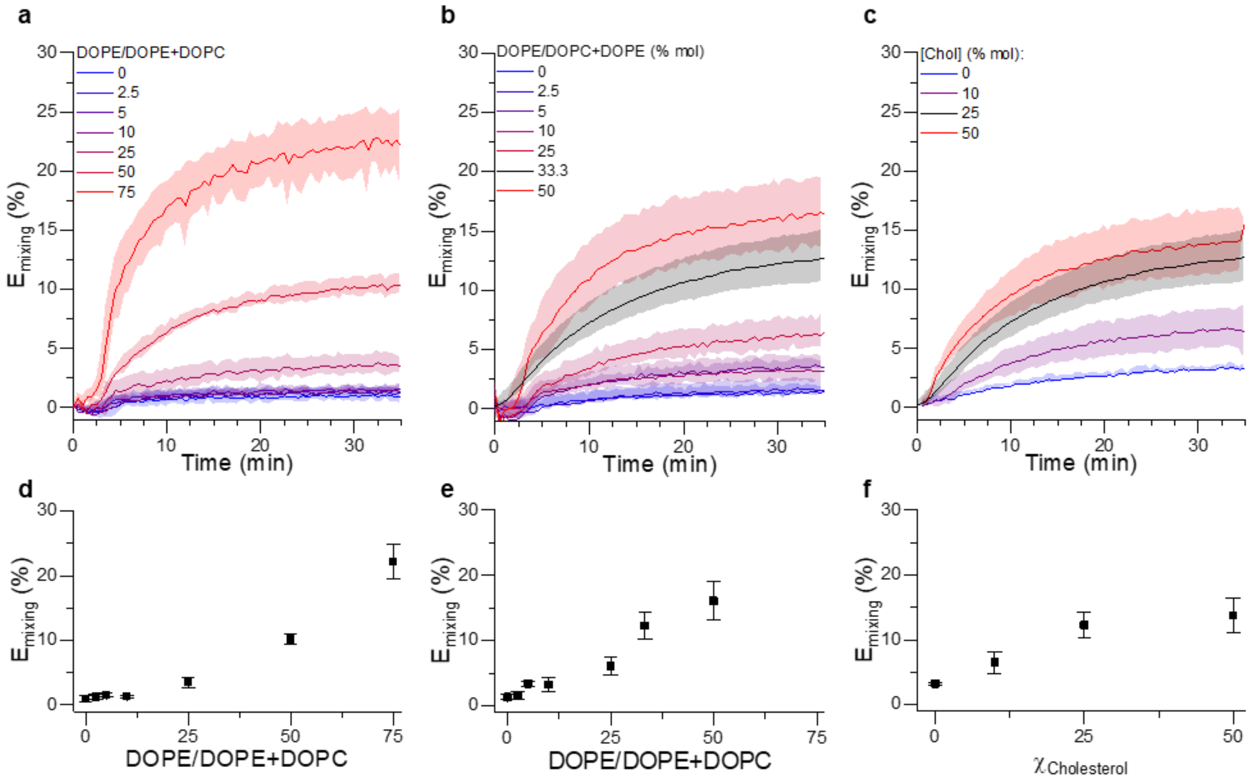

Fig. S4 | Effect of DOPE and cholesterol concentration on fusion-induced lipid mixing efficiency. a-c) Kinetic traces for E_{mixing} and **d-f)** E_{mixing} at 35 minutes. for a,d) *L* LUVs with a χ_{DOPE}/1-χ_{DOPE} composition; b,e) 50-χ_{DOPE}/χ_{DOPE}/25 DOPC/DOPE/Chol composition and c,f) 50-0.5χ_{Chol}/ 25-0.5χ_{Chol}/χ_{Chol} composition. Composition of all *U* LUVs was 50/25/25 DOPC/DOPE/Chol.

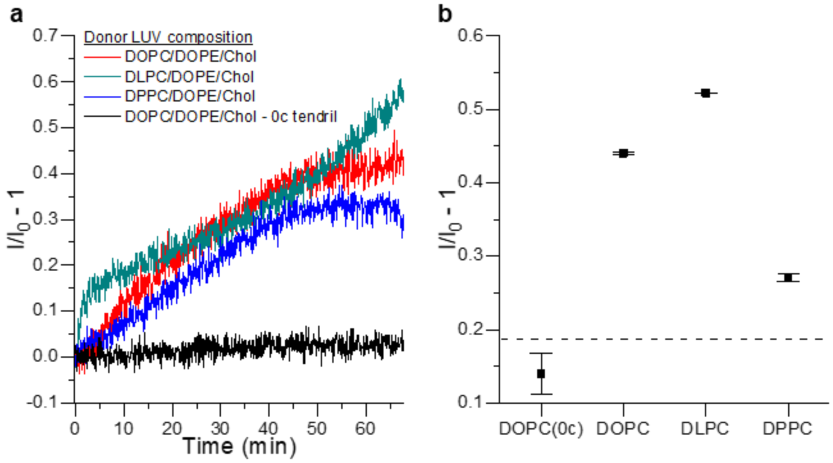

Fig. S5 | Tb/DPA content mixing assay. a) Kinetic traces showing the increase of DPA fluorescence (I_{544}) upon incubation of *L* vesicles of varied composition with DOPC/DOPE/Chol *U* liposomes. **b)** Change in DPA fluorescence intensity as a function of the lamellar lipid of the donor vesicle, calculated from the 490nm and 544nm DPA emission peaks. Symbols represent the mean, error bars the standard deviations of the intensity change corresponding to the two emission bands.

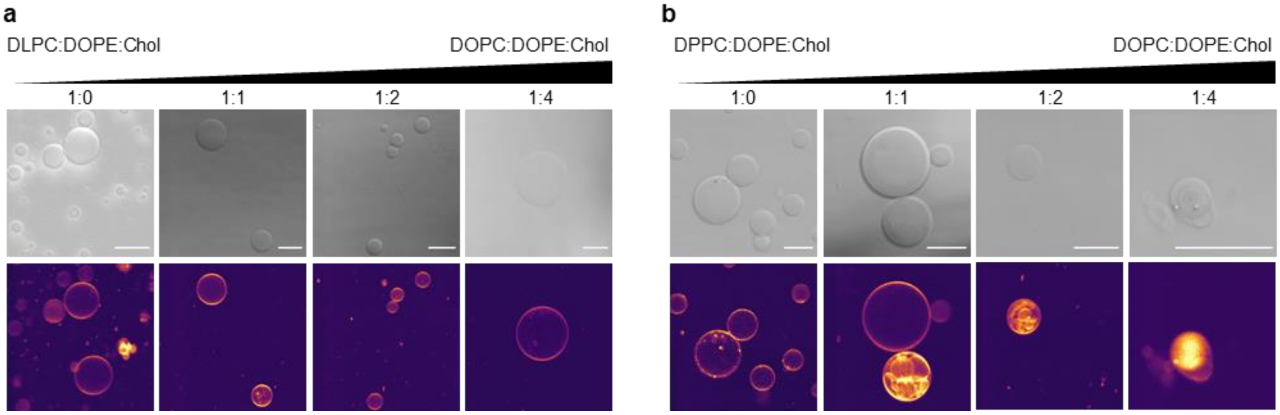

Fig. S6 | Microscopy characterization of anomalous fusion products. a) Brightfield and confocal microscopy images of GUVs at increasing DLPC/DOPE/Chol to DOPC/DOPE/Chol ratio. **b)** Brightfield and confocal microscopy images of GUVs at increasing DPPC/DOPE/Chol to DOPC/DOPE/Chol ratio. Scalebar: 20µm. See Table S4 for detailed composition.

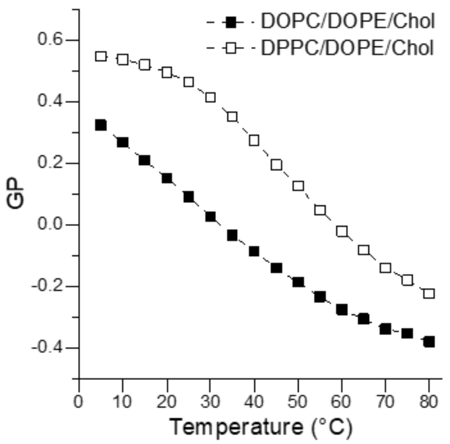

Fig. S7 | Change in Laurdan GP at increasing temperature. The change in gradient observed for the DPPC/DOPE/Chol composition suggests a L_o → L_α transition, while not such change is observed for DOPC/DOPE/Chol membranes, indicating they are in the L_α phase at 25°C.

Supplementary Table S1: DNA sequences

Name	Sequence (5'→3')
T_{L1}	GCCACTTCACCCGACCATTCTGGCCGTTGCGCTCGTGAAAGTAGC
T_{L1}_chol3	GCCACTTCACCCGACCATTCTGGCCGTTGCGCTCGTGAAAGTAGC-TEG-Cholesteryl
T_{L2}	CGAGTAAGGGCGAGCGCAACGGCCAGAATGGTCGGTCACTCAAAC
T_{L2}_chol5	Cholesteryl-TEG-CGAGTAAGGGCGAGCGCAACGGCCAGAATGGTCGGTCACTCAAAC
T_{U1}	GCTACTTTCACGAGCGCAACGGCCAGAATGGTCGGGTGAAGTGGC
T_{U1}_chol5	Cholesteryl-TEG-GCTACTTTCACGAGCGCAACGGCCAGAATGGTCGGGTGAAGTGGC
T_{U2}	GTTTGAGTGACCGACCATTCTGGCCGTTGCGCTCGCCCTTACTCG
T_{U2}_chol3	GTTTGAGTGACCGACCATTCTGGCCGTTGCGCTCGCCCTTACTCG-TEG-Cholesteryl
db-T_L (0)	GTTTGAGTGACCGGGTGAAGTGGC
db-T_U (0)	GCCACTTCACCCGGTCACTCAAAC
db-T_L (4)	ATGAGTTTGAGTGACCGGGTGAAGTGGCTGCT
db-T_U (4)	AGCAGCCACTTCACCCGGTCACTCAAACTCAT
db-T_L (6)	GGATGAGTTTGAGTGACCGGGTGAAGTGGCTGCTGT
db-T_U (6)	ACAGCAGCCACTTCACCCGGTCACTCAAACTCATCC
db-T_L (8)	GCGGATGAGTTTGAGTGACCGGGTGAAGTGGCTGCTGTCT
db-T_U (8)	AGACAGCAGCCACTTCACCCGGTCACTCAAACTCATCCGC
db-T_L (10)	CTGCGGATGAGTTTGAGTGACCGGGTGAAGTGGCTGCTGTCTGG
db-T_U (10)	CCAGACAGCAGCCACTTCACCCGGTCACTCAAACTCATCCGCAG
db-T_L (12)	TTCTGCGGATGAGTTTGAGTGACCGGGTGAAGTGGCTGCTGTCTGGAG
db-T_U (12)	CTCCAGACAGCAGCCACTTCACCCGGTCACTCAAACTCATCCGCAGAA
b-T_L	GTTTGAGTGACCGGGTGAAGTGGCTGCTGTCTGGAG
ub-T_L	CTCCAGACAGCAGCCACTTCACCCGGTCACTCAAAC

Supplementary Table S2: DNA sequences used to assemble each tendril

Name	DNA strands
Standard tendrils	
T_L – 0c	T_{L1}, T_{L2}
T_L – 1c unzipped	T_{L1}-chol3, T_{L2}
T_L – 1c zipped	T_{L1}-chol3, T_{L2}
T_L – 2c (T_L unless otherwise stated)	T_{L1}-chol3, T_{L2}-chol5
T_U – 0c	T_{U1}, T_{U2}
T_U – 1c unzipped	T_{U1}, T_{U2}-chol3
T_U – 1c zipped	T_{U1}-chol5, T_{U2}
T_U – 2c (T_U unless otherwise stated)	T_{U1}-chol5, T_{U2}-chol3
Delayed tendrils	
Delayed T_L	T_{L1}-chol3, T_{L2}-chol5, db-T_L (n)
Delayed T_U	T_{U1}-chol5, T_{U2}-chol3, db-T_U (n)
Blocked tendrils	
Blocked T_L	T_{L1}-chol3, T_{L2}-chol5, bT_L

Supplementary Table S3: Lipid composition of the LUVs used throughout the experiments.

ID	Lamellar Lipid		Fusogenic Lipid		Sterol		Notes
	Lipid Name	Molar fraction	Lipid Name	Molar fraction	Lipid Name	Molar fraction	
DOPE/DOPC ratio effect, no cholesterol							
A0	DOPC	100	DOPE	0	Cholesterol	0	
A1	DOPC	97.5	DOPE	2.5	Cholesterol	0	
A2	DOPC	95	DOPE	5	Cholesterol	0	
A3	DOPC	90	DOPE	10	Cholesterol	0	
A4	DOPC	75	DOPE	25	Cholesterol	0	
A5	DOPC	50	DOPE	50	Cholesterol	0	
A6	DOPC	25	DOPE	75	Cholesterol	0	

Effect of DOPE/DOPC ratio effect, 25%mol cholesterol							
B0	DOPC	75	DOPE	0	Cholesterol	25	
B1	DOPC	73.125	DOPE	1.875	Cholesterol	25	
B2	DOPC	71.25	DOPE	3.75	Cholesterol	25	
B3	DOPC	67.5	DOPE	7.5	Cholesterol	25	
B4	DOPC	56.25	DOPE	18.75	Cholesterol	25	
B5	DOPC	37.5	DOPE	37.5	Cholesterol	25	
Cholesterol effect, 1:2 DOPE/DOPC ratio							
C0	DOPC	66.67	DOPE	33.33	Cholesterol	0	
C1	DOPC	60	DOPE	30	Cholesterol	10	
C2	DOPC	50	DOPE	25	Cholesterol	25	Reference, used for *U* LUVs
C3	DOPC	33.33	DOPE	16.67	Cholesterol	50	
Lamellar lipid effect							
D1	DLPC	50	DOPE	25	Cholesterol	25	
D2	DMPC	50	DOPE	25	Cholesterol	25	
D3	DPPC	50	DOPE	25	Cholesterol	25	
D4	DSPC	50	DOPE	25	Cholesterol	25	
D5	DAPC	50	DOPE	25	Cholesterol	25	
D6	POPC	50	DOPE	25	Cholesterol	25	
D7	DPhPC	50	DOPE	25	Cholesterol	25	Required Freeze-Thaw
D8	DOPC	50	DOPE	25	Cholesterol	25	Reference, used for *U* LUVs
D9	DEPC	50	DOPE	25	Cholesterol	25	
Fusogenic lipid effect							
E1	DOPC	50	DOPE	25	Cholesterol	25	Reference, used for *U* LUVs
E2	DOPC	37.5	OA	37.5	Cholesterol	25	1:1 ratio of DOPC to OA to correct for the number of HC chains. Required Freeze-Thaw
E3	DOPC	50	POPA	25	Cholesterol	25	
E4	DOPC	50	POPE	25	Cholesterol	25	
E5	DOPC	50	DOPC	25	Cholesterol	25	

Supplementary Table S4: Lipid composition of the GUVs and XRD samples.

ID	Lamellar Lipid		Fusogenic Lipid		Sterol		Notes
	Lipid Name	Molar fraction	Lipid Name	Molar fraction	Lipid Name	Molar fraction	
G0	DLPC	50	DOPE	25	Cholesterol	25	
G1	DLPC/DOPC	25/25	DOPE	25	Cholesterol	25	
G2	DLPC/DOPC	16.67/33.33	DOPE	25	Cholesterol	25	
G3	DLPC/DOPC	12.5/37.5	DOPE	25	Cholesterol	25	
G4	DLPC/DOPC	10/40	DOPE	25	Cholesterol	25	
G5	DOPC	50	DOPE	25	Cholesterol	25	No GUVs formed
G6	DPPC	50	DOPE	25	Cholesterol	25	
G7	DPPC/DOPC	25/25	DOPE	25	Cholesterol	25	
G8	DPPC/DOPC	16.67/33.33	DOPE	25	Cholesterol	25	
G9	DPPC/DOPC	12.5/37.5	DOPE	25	Cholesterol	25	
G10	DPPC/DOPC	10/40	DOPE	25	Cholesterol	25	

References:

1 C. François-Martin and F. Pincet, *Sci. Rep.*, 2017, **7**, 43860.

2 A. J. Smith, S. G. Alcock, L. S. Davidson, J. H. Emmins, J. C. Hiller Bardsley, P. Holloway, M. Malfois, A. R. Marshall, C. L. Pizzey, S. E. Rogers, O. Shebanova, T. Snow, J. P. Sutter, E. P. Williams and N. J. Terrill, *J. Synchrotron Radiat.*, 2021, **28**, 939–947.

3 N. Kučerka, M. P. Nieh and J. Katsaras, *Biochim. Biophys. Acta - Biomembr.*, 2011, **1808**, 2761–2771.

4 N. Kučerka, Y. Liu, N. Chu, H. I. Petrache, S. Tristram-Nagle and J. F. Nagle, *Biophys. J.*, 2005, **88**, 2626–2637.

5 W. J. Sun, S. Tristram-Nagle, R. M. Suter and J. F. Nagle, *Biophys. J.*, 1996, **71**, 885–891.

6 B. Kollmitzer, P. Heftberger, M. Rappolt and G. Pabst, *Soft Matter*, 2013, **9**, 10877–10884.

7 E. E. Kooijman, V. Chupin, N. L. Fuller, M. M. Kozlov, B. De Kruijff, K. N. J. Burger and P. R. Rand, *Biochemistry*, 2005, **44**, 2097–2102.
